# Factors influencing prosthesis selection and variation: a national survey of orthopaedic surgeons in Ireland

**DOI:** 10.1007/s11845-026-04328-9

**Published:** 2026-04-14

**Authors:** Conor Farrell, Conor Kilkenny, Imad Mirza, John F. Quinlan

**Affiliations:** 1 Tallaght University Hospital, Tallaght, Dublin 24, Ireland; 2 Royal College of Surgeons in Ireland, Dublin 2, Ireland

## Introduction

Total hip arthroplasty (THA) and total knee arthroplasty (TKA) are among the most performed and clinically successful orthopaedic procedures, particularly in the management of end-stage osteoarthritis. These operations provide substantial improvements in pain relief, mobility and quality of life for patients with degenerative joint disease [1]. In Ireland, over 7,000 primary THA and TKA procedures are performed annually in the public sector alone, with demand projected to rise [2]. Osteoarthritis affects approximately 12.9% of adults and more than 20% of those aged over 80 years [3]. Subsequently, upward trends of arthroplasty are evident internationally; between 2011 and 2021, hip and knee arthroplasty volumes rose by 15% and 19% respectively across OECD countries [3].

Despite their clinical benefits, THA and TKA are among the most resource-intensive procedures in modern healthcare. Implant costs alone may account for up to 60% of the total procedural expenditure [4]. Implant costs vary substantially between manufacturers and designs, while registry-based evidence suggests that higher-cost implants do not consistently demonstrate superior long-term clinical outcomes or lower revision rates at a population level [4–6] In Ireland, they are consistently listed among the highest-cost interventions performed in public hospitals [7]. With a global shift toward value-based care, there is growing emphasis on balancing implant quality, long-term outcomes, and fiscal responsibility. The decisions orthopaedic surgeons make in selecting prostheses have direct implications for patient outcomes, revision rates, and healthcare system sustainability [8].

Failures in implant selection, due to unfamiliarity, limited cost transparency, or a lack of reported outcome data on implants can contribute to increased morbidity and unnecessary revision surgeries, thus imposing further strain on healthcare resources. National arthroplasty registries have emerged as critical tools in evaluating implant performance and guiding safer prosthesis selection. Countries such as Sweden, Australia, and the UK have demonstrated that registry engagement can lead to reduced revision rates, greater standardisation in care, and earlier identification of underperforming implants [4, 9, 10].

In Ireland, the Irish National Orthopaedic Register (INOR) was established in 2014 with the aim of enabling data-driven quality assurance and outcome monitoring [1]. However, the extent to which Irish orthopaedic surgeons actively consult or utilise registry data to inform their clinical decisions remains unclear.

A further challenge is the persistent variation in implant choice observed across surgeons, institutions, and regions. While some degree of variation is appropriate, reflecting differences in patient anatomy, comorbidities, or activity level, variation may also arise from surgeon-related factors rather than patient need. International studies suggest that implant familiarity, perceived quality, autonomy, and training background frequently outweigh cost or registry considerations when selecting prostheses [5, 8].

Irish Orthopaedic consultants, especially in the private sector, report considerable autonomy in implant choice, while trainees are often excluded from such decisions, limiting their exposure to cost-conscious and evidence-based selection frameworks. Although prosthesis procurement in Ireland is governed by national frameworks designed to promote consistency and cost-effectiveness, their influence on individual clinician behaviour remains poorly understood [8, 9]. Additionally, in the private sector, oversight and standardisation are less robust.

A lack of formal education in procurement practices, implant cost-awareness, and the principles of value-based care, particularly among junior doctors, may contribute to entrenched patterns of behaviour with a reluctance to adopt to novel innovations. Without early and structured exposure to these non-clinical competencies, future orthopaedic consultants may continue to make implant choices driven by habit rather than evidence or cost-effectiveness.

To date, there has been no national study evaluating the drivers of prosthesis selection among orthopaedic surgeons in Ireland. While registry data can offer insight into implant performance and utilisation trends, it does not explain the underlying rationale guiding individual surgeon decisions. A deeper understanding of these drivers, including awareness of cost, use of registry data, perceived implant quality, and departmental consensus is essential for informing procurement policy, medical education, and the future direction of orthopaedic practice in Ireland. This study seeks to address these knowledge gaps by evaluating the key factors that influence prosthesis selection in hip and knee arthroplasty and examining how these factors vary by surgeon grade, hospital type, and region. The findings aim to support more standardised, evidence-informed implant practices while also promoting responsible and sustainable resource use in arthroplasty.

## Methods

### Study design and participants

This was a national cross-sectional survey of orthopaedic surgeons in Ireland designed to identify factors influencing prosthesis selection in total hip arthroplasty (THA) and total knee arthroplasty (TKA), and to explore variation in implant choice across hospital settings, regions, and levels of surgical experience. Eligible participants included consultant orthopaedic surgeons and orthopaedic specialist registrars practising in Ireland, in both public and private sectors.

Surgeons were identified through professional bodies, including the Irish Institute of Trauma and Orthopaedic Surgery (ITTOS), national training scheme directories, hospital group staff lists, and professional networks. Invitations were distributed to 163 consultant orthopaedic surgeons and 65 orthopaedic specialist registrars nationally.

### Survey instrument

The survey instrument was developed following review of a previously published international study examining factors influencing prosthesis selection, implant variation, and procurement practices in hip and knee arthroplasty. Survey domains were adapted from these studies and refined by the authors to ensure relevance to the Irish healthcare context. The draft survey was piloted informally among a small group of orthopaedic surgeons to assess clarity, face validity, and completion time, with minor modifications made prior to dissemination.

The survey collected information on surgeon demographics, arthroplasty workload, autonomy in implant selection, cost awareness, decision-making factors influencing implant choice, and engagement with national joint registry data. For the purposes of this study, *cost* was defined as the direct acquisition cost of the implant to the healthcare provider, excluding theatre time, staffing, or downstream care costs. *Value-based healthcare* was conceptualised as achieving optimal clinical outcomes relative to cost.

### Data collection and ethics

The survey was open for a six-week period from July 2025 to mid-August 2025. A total of 228 surgeons were invited to participate. Invitations were distributed via Irish Orthopaedic Association mailing lists, hospital group email distribution systems, professional communication platforms, and through the Royal College of Surgeons in Ireland. Reminder messages were issued at two-week intervals.

Participation was voluntary and anonymous, and informed consent was implied by completion of the survey.

## Results

### Respondent demographics

**Fig. 1:**
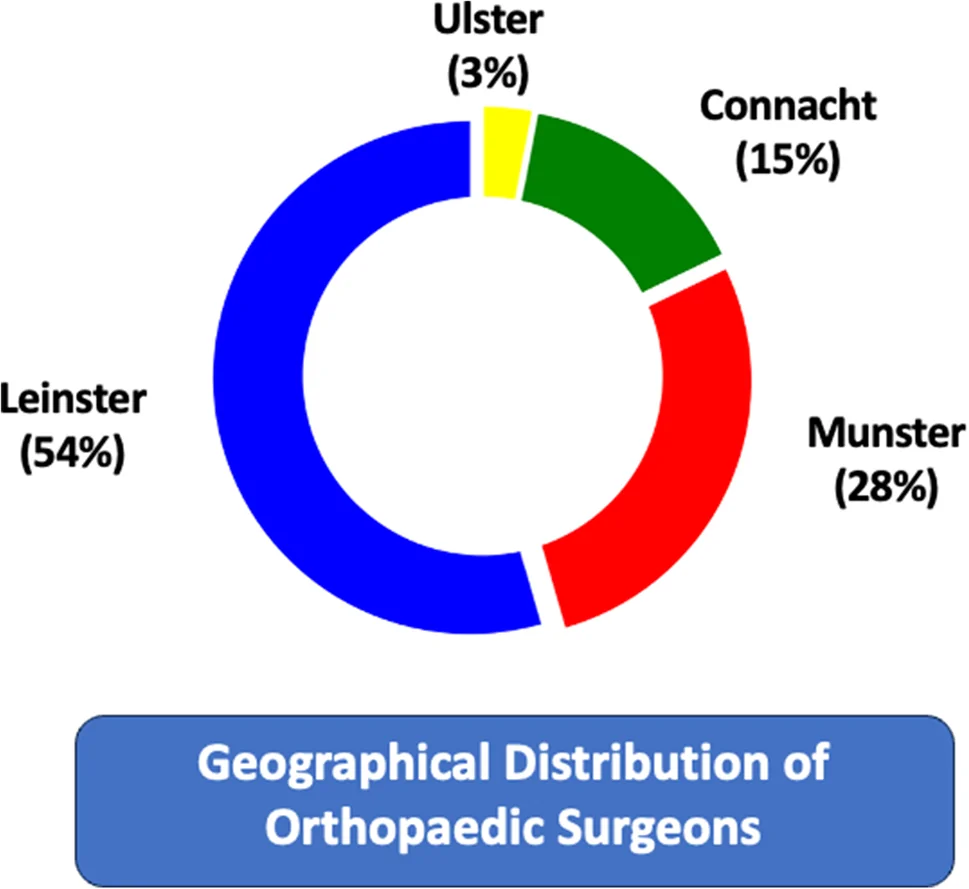
Geographical distribution of orthopaedic surgeons

**Fig. 2:**
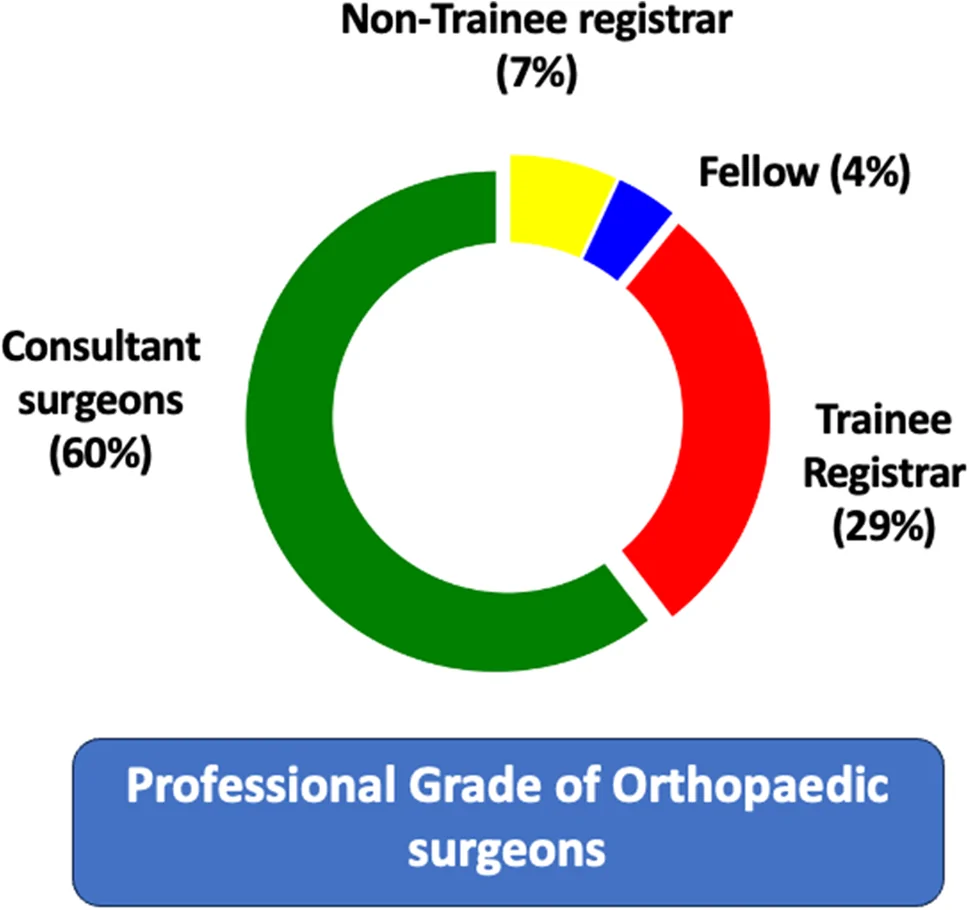
Professional grade of orthopaedic surgeons

A total of 228 orthopaedic surgeons were invited to participate, with 101 respondents completing the survey, giving an overall response rate of 44.3%. The geographical distribution of respondents reflected the national spread of orthopaedic services, with the majority practising in Leinster (54%), followed by Munster (28%), Connacht (*n* = 15%), and Ulster (3.0%) (Fig. 1). Regarding professional grade, 61 respondents (60.4%) identified as consultant orthopaedic surgeons, while 29 (28.7%) were training registrars, 7 (6.9%) were non-training registrars, and 4 (4.0%) were clinical fellows (Fig. 2).

**Fig. 3:**
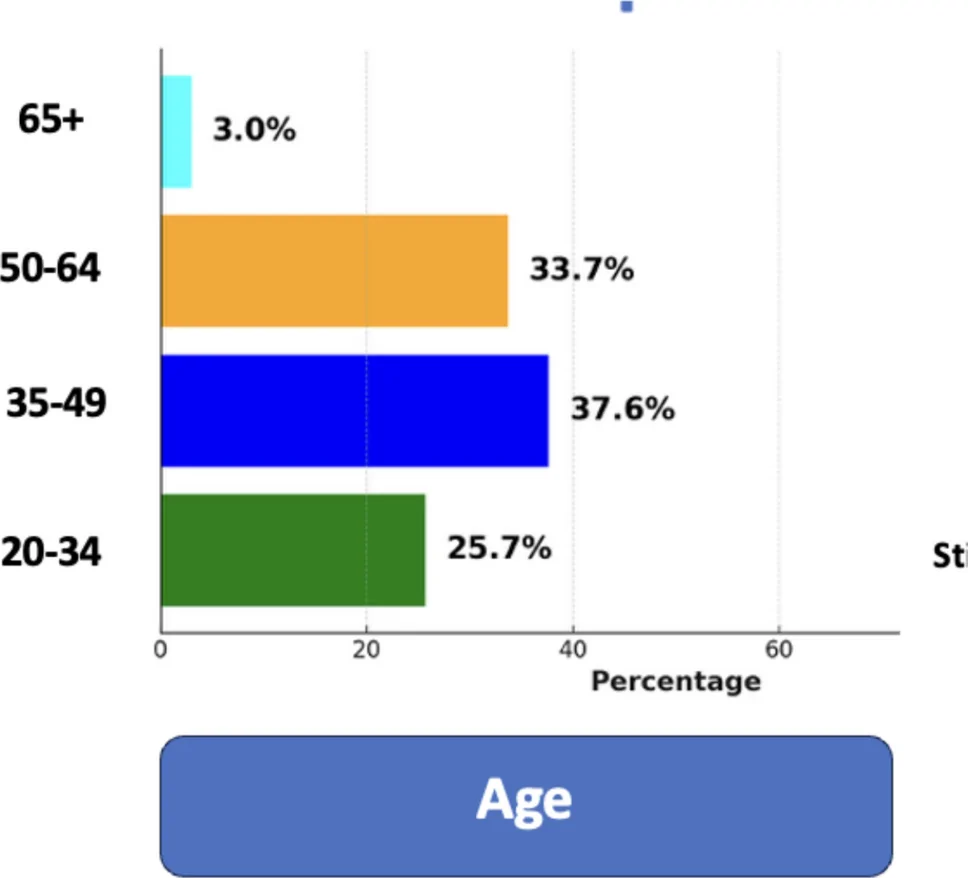
Age of orthopaedic surgeons

The age distribution was concentrated in the middle career stages, with the majority of respondents aged 35–49 years (*n* = 38, 37.6%) and 50–64 years (*n* = 34, 33.7%). A smaller number were aged 20–34 years (*n* = 26, 25.7%) or 65 years and older (*n* = 3, 3%) (Fig. 3).

**Fig. 4:**
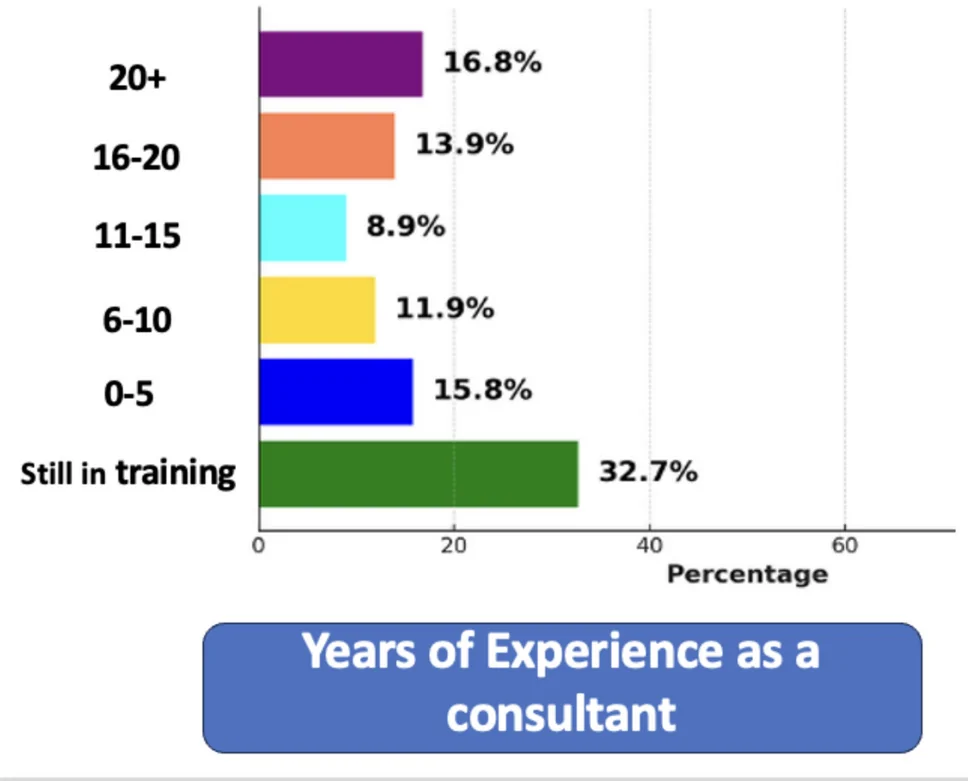
Years of experience as an orthopaedic surgeon

Experience levels were varied, with the majority, 32.7% still in training. 16.8% of respondents had more than 20 years of post-training experience. The remainder were distributed across the 0–5 years (15.8%), 6–10 years (11.9%), 11–15 years (8.9%), and 15–20 years (13.9%) categories (Fig. 4).

### Practice type and workload

The majority of respondents (*n* = 96, 96.0%) reported performing both total hip arthroplasty (THA) and total knee arthroplasty (TKA). A small number specialised in THA only (*n* = 2, 2%) or TKA only (*n* = 2, 2%) (Table 1.)

**Table 1:** Practice type

Procedure Type	Number of Respondents	Percentage (%)
THA + TKA	96	96
THA only	2	2
TKA only	2	2

**Fig. 5:**
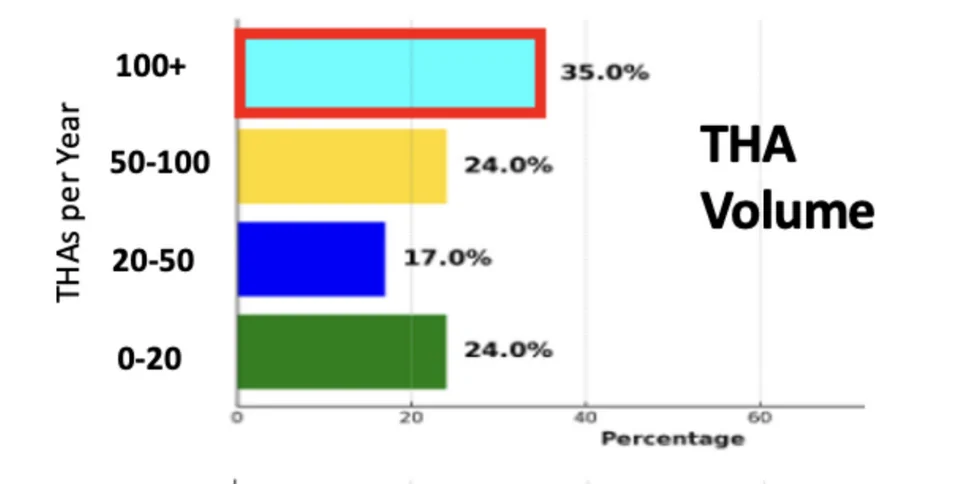
Total hip arthroplasty volume

Surgical volume varied across respondents. For THA, of the one hundred respondents, 35 surgeons (35%) reported performing more than 100 procedures per year, 24% performed 50–100 annually, 17% performed 20–50, and 24% performed fewer than 20 (Fig. 5).

**Fig. 6:**
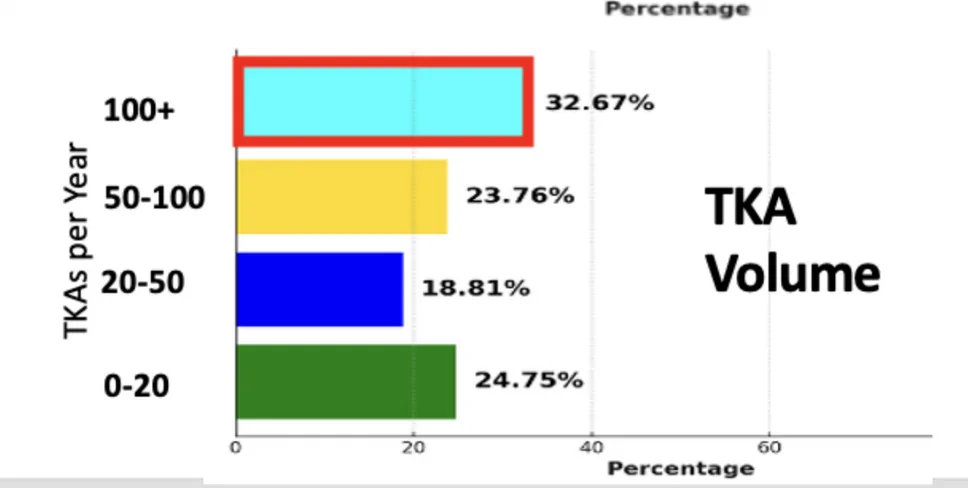
Total knee arthroplasty volume

Similarly, for TKA, of the 101 respondents, 33 surgeons (32.67%) reported more than 100 annual procedures, 23 (23.76%) performed 50–100, 19 (18.81%) performed 20–50, and 25 (24.75%) carried out fewer than 20 procedures annually (Fig. 6).

**Fig. 7:**
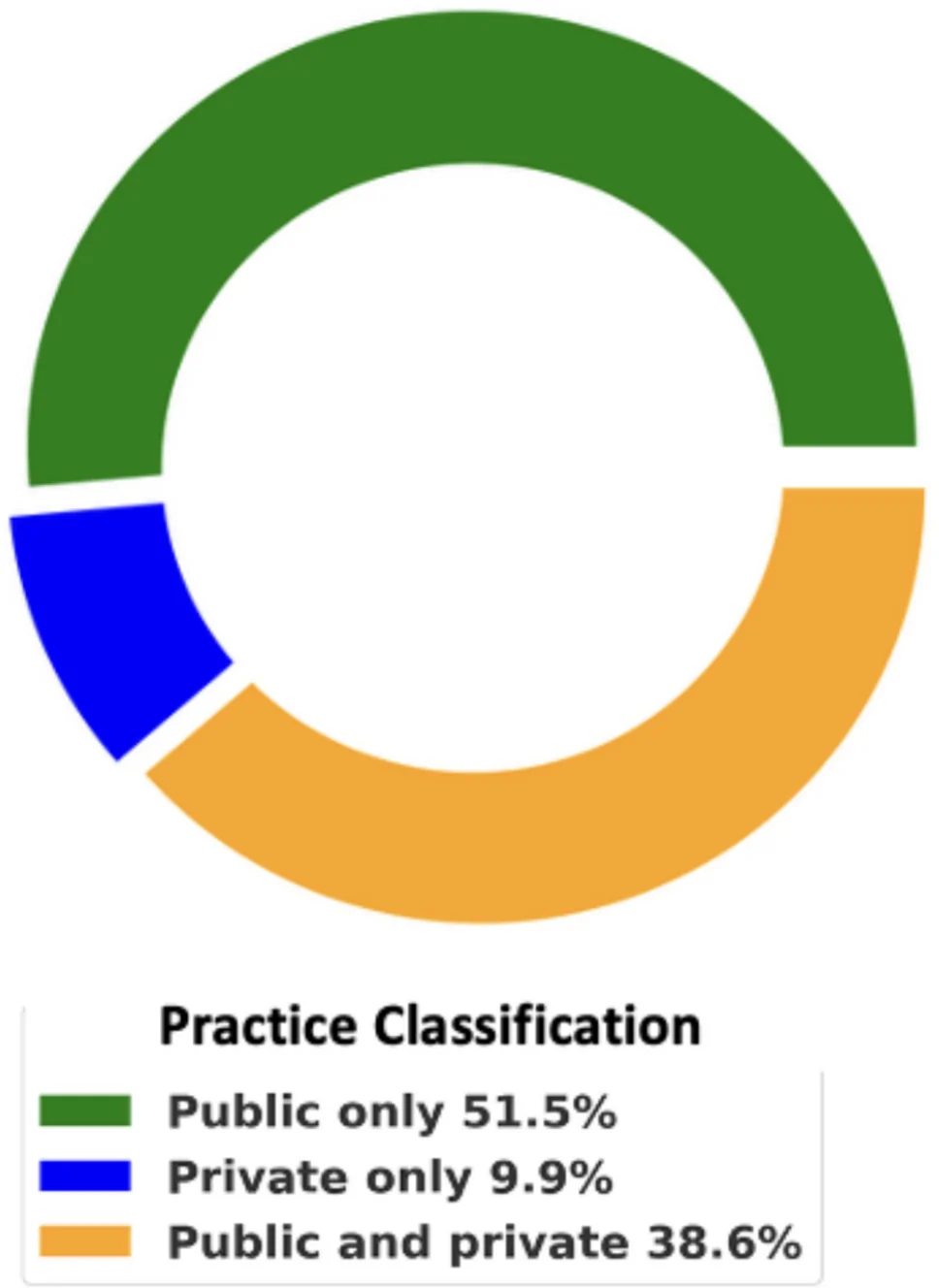
Practice classification

Practice setting was broadly distributed: 39 surgeons (38.6.0%) reported working across both public and private sectors, 52 (51.5%) worked exclusively in the public sector, and 10 (9.9%) worked only in private practice (Fig. 7).

### Autonomy in implant selection

Levels of autonomy in implant selection varied according to both clinical setting and surgeon seniority. Among consultants who responded to the relevant question, 45 out of 54 (83.3%) reported having full autonomy over implant choice in the public sector. Seven consultants (13%) described departmental consensus as the prevailing decision-making model, while two respondent (3.7%) reported limited input. In the private sector, autonomy was even more pronounced, with 50 of 51 consultants (98%) reporting full autonomy, one (2%) reporting departmental consensus (Table 2).

**Table 2:** Consultant autonomy in public and private hospitals

Setting	Decision-Making Model	Number of Consultants	Percentage (%)
Public Sector	Full Autonomy	45	83.3
Public Sector	Departmental Consensus	7	13
Public Sector	Limited Input	2	3.7
Private Sector	Full Autonomy	50	98
Private Sector	Departmental Consensus	1	2

In contrast, registrars reported significantly less influence. Six of the forty three registrars (14%) who answered the public sector autonomy question reported full autonomy. Eleven (26%) cited departmental consensus, 19 (44%) reported limited input, and 7 (16%) selected “other.” Which described the consultants as the ones choosing. (Table 3) A similar trend was observed in the private sector, where 3 of the 26 responding registrars reported full autonomy(11.5%); Five (19.2%) reported limited input, 2 (19.2%) cited departmental consensus, and 16 (61.5%) selected “other.” (Table 4).

**Table 3:** Registrar autonomy in public hospitals

Autonomy Level	Number of Registrars	Percentage (%)
Full Autonomy	6	14
Departmental Consensus	11	26
Limited Input	19	44
Other	7	16

**Table 4:** Registrar autonomy in private hospitals

Autonomy Level	Number of Registrars	Percentage (%)
Full Autonomy	3	11.5
Departmental Consensus	2	7.7
Limited Input	5	19.2
Other	16	61.6

### Cost awareness

**Fig. 8:**
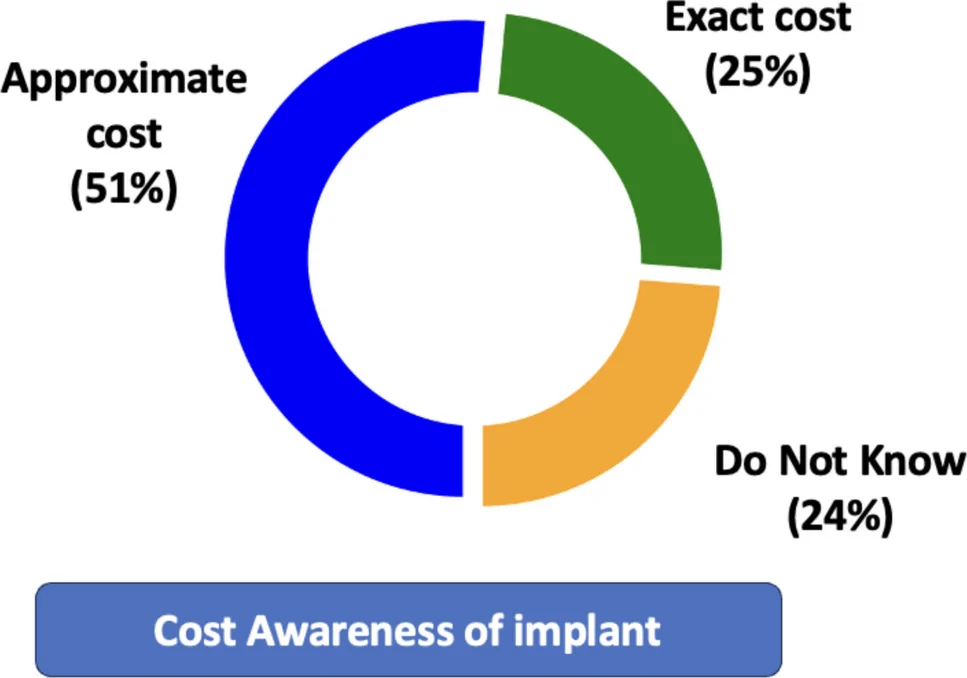
Cost awareness of implant

Respondents demonstrated varying levels of awareness regarding the cost of the implants they used most frequently. Overall, 52 participants (51%) reported knowing the approximate cost (within €1,000), 25 (25%) reported knowing the exact cost, while 24 (24%) indicated they did not know the cost (Fig. 8).

Clear differences emerged between consultants and registrars. Among consultants, 59.0% reported knowing the approximate cost and 34.4% reported knowing the exact cost and 6.5% did not know the cost. In contrast, the majority (50%) of registrars stated they did not know the cost of the implants they used. 40% reported knowing the approximate cost and 10% reported knowing the exact cost (Table 5).

**Table 5:** Cost awareness between consultants and registrars

Cost Awareness	Consultants (*n* = 61)	Registrars (*n* = 40)
Exact Cost	34.40%	10.00%
Approximate Cost	59.00%	40.00%
Do Not Know	6.50%	50.00%

### Factors influencing implant selection

**Fig. 9:**
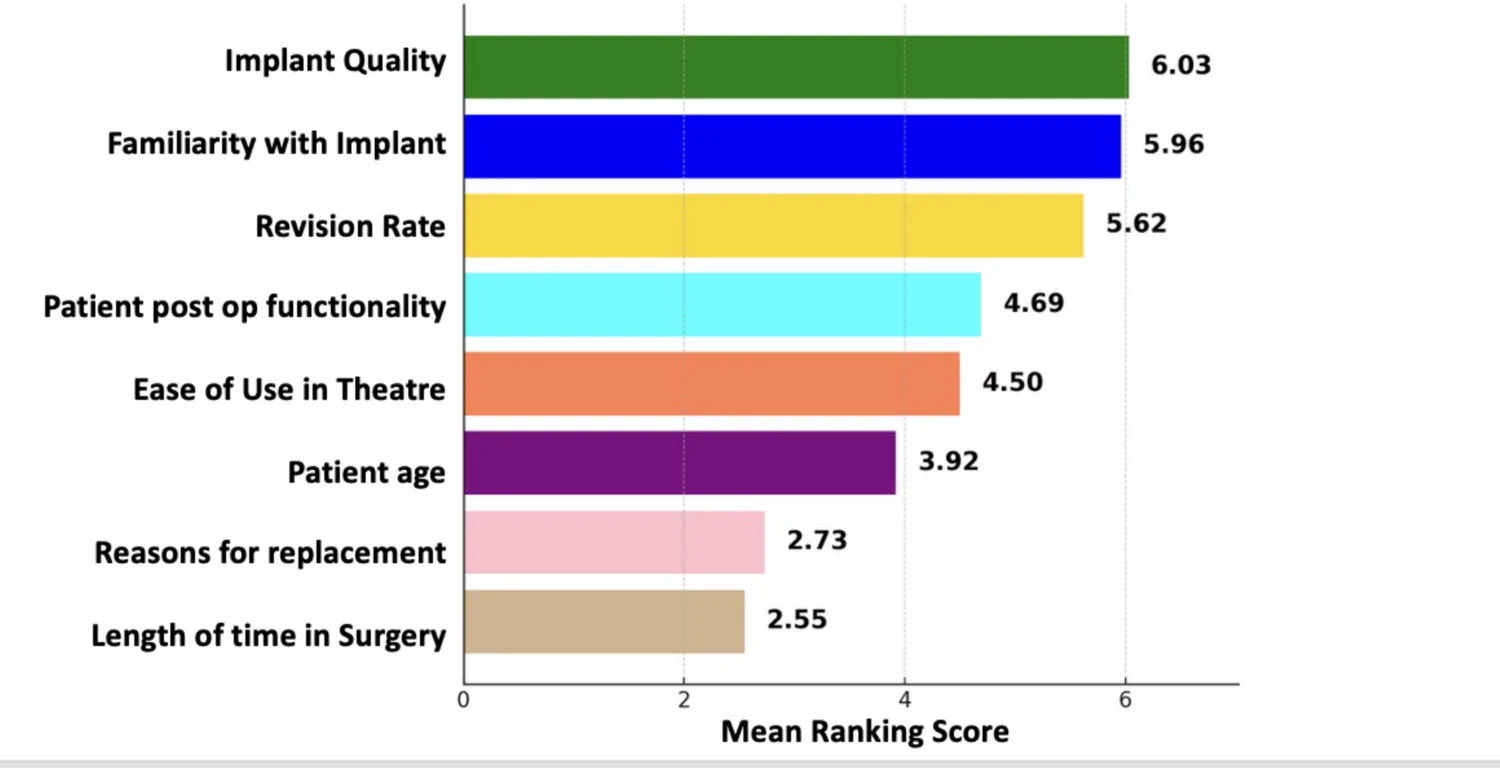
Factors influencing prosthesis selection

The most influential factor was implant quality, with a mean score of (6.03), followed closely by familiarity with implant (5.96) and revision rate (5.62). Post-operative functionality (mean score 4.69) and ease of use in theatre (4.5) were rated as moderately important. In contrast, patient age (3.92), reasons for replacement (2.73), and length of time in surgery (2.55) were generally considered less influential (Fig. 9).

### Registry engagement and importance

**Fig. 10:**
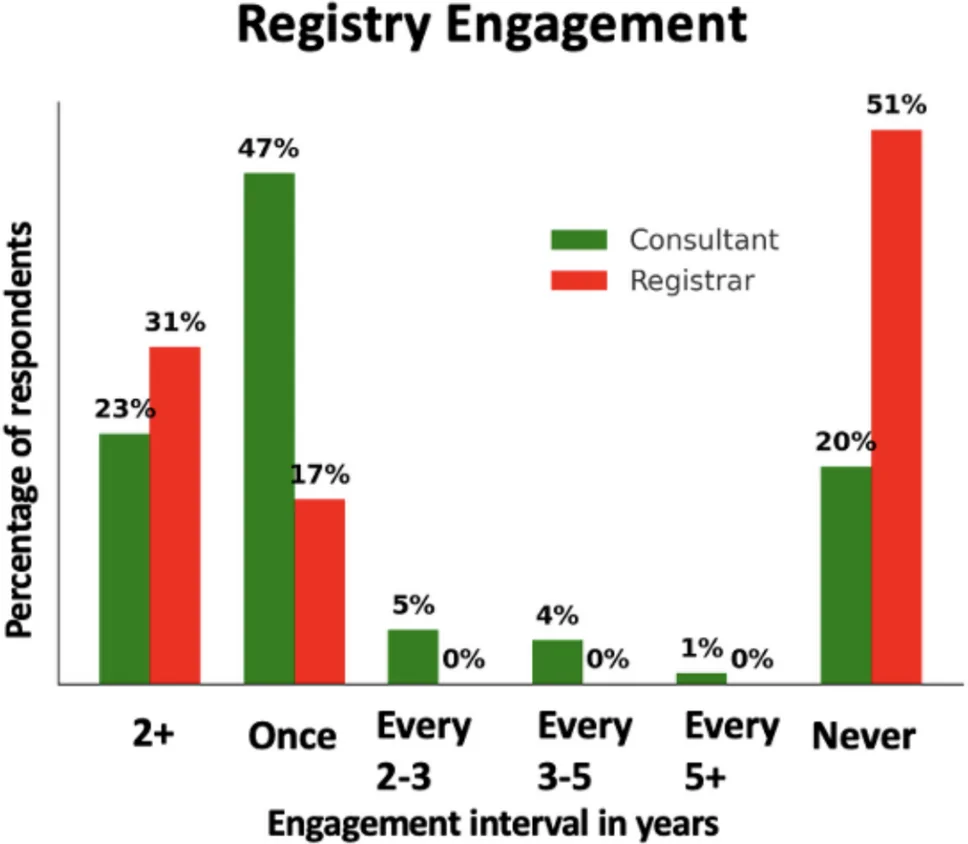
Frequency of registry engagement

Surgeons reported varying frequencies of engaging with national joint registry data. Most Consultant respondents (46.7%) stated they check registry results once a year, while 23.3% reviewed data multiple times annually. A smaller proportion checked data every 2–3 years (5%), every 3–5 years (4%), or every 5 + years (1.6%), and 20% never consulted registry results. Registrar respondents (17.1%) stated they check registry results once a year, while 31.4% reviewed data multiple times annually. No registrars checked data every 2–3 years, every 3–5 years, or every 5 + years. The majority of registrars, 51.4% never consulted registry results (Fig. 10).

**Fig. 11:**
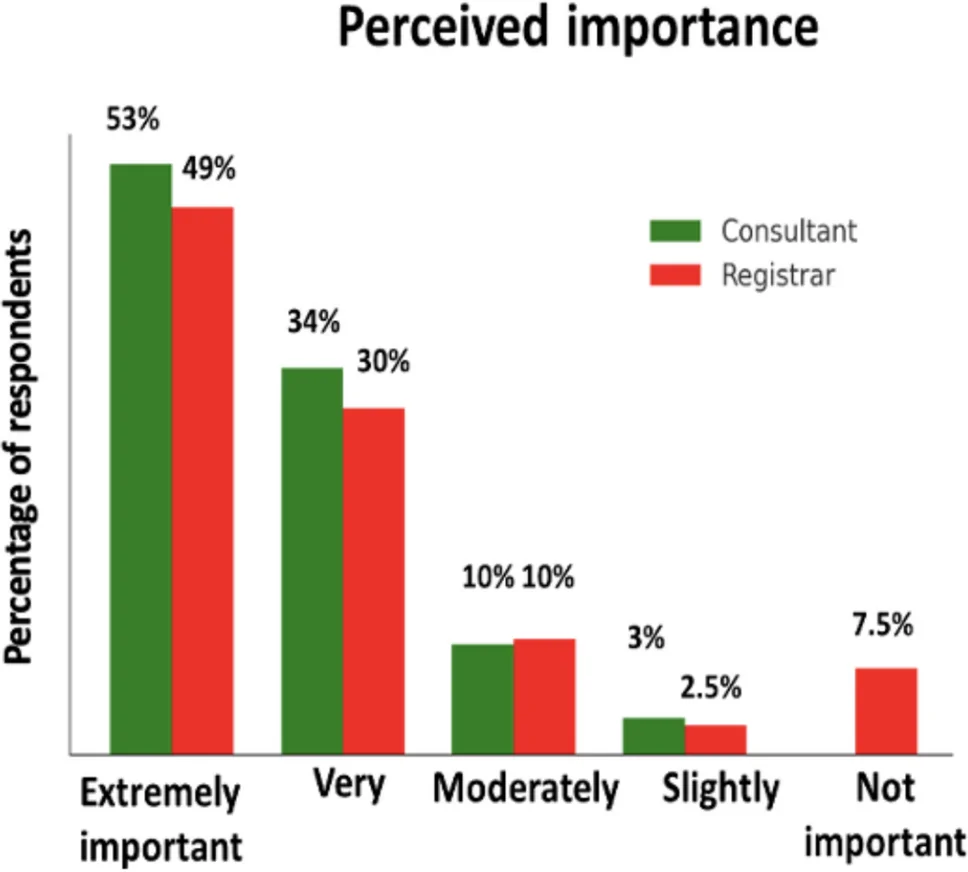
Perceived importance of registry engagement

Participants were also asked to rate the importance of registry data in their clinical decision-making. Among consultants (*n* = 61), 32 (52.5%) reported that registry outcomes were *extremely important*, 21 (34.4%) rated them as *very important*, while smaller proportions considered them *moderately important* (*n* = 9.8%), *slightly important* (*n* = 2, 3.3%), or *not important* (0%). Among registrars (*n* = 39), 19 (48.7%) considered registry outcomes *extremely important*, 12 (30.7%) *very important*, 4 (10.3%) *moderately important*, 1 (2.6%) *slightly important*, and 3 (7.7%) *not important* (Fig. 11).

## Discussion

This national survey provides new insights into the factors influencing prosthesis selection among Irish orthopaedic surgeons. Our findings align with international evidence suggesting that surgeon decision-making is primarily driven by implant quality, familiarity, and revision rates, with cost playing a secondary role [1, 10]. Despite the availability of national procurement frameworks and registry data, substantial variation remains in implant choice, autonomy, and cost awareness, particularly across grades and sectors.

Consistent with prior studies, implant familiarity was one of the highest-rated influences on prosthesis selection [1, 5]. While this may reflect confidence in outcomes, it also raises concerns about conservatism in implant choice and resistance to change, even in the presence of emerging evidence or cost-effective alternatives [4]. Sharkey et al. highlighted that surgeons often continue using the same implants despite viable alternatives, citing a reluctance to undergo new learning curves [5]. Similar patterns have been reported in oral and maxillofacial surgery, where perceived autonomy and implant familiarity strongly shape selection even when institutional guidelines recommend otherwise [11]. Ongoing education and regular review of up-to-date implant performance data are essential to ensure that surgeons remain responsive to evolving evidence, preventing the continued use of suboptimal implants simply because they are familiar or convenient.

In our study, revision rate was the third most influential factor in implant selection, indicating its significance in decision-making but secondary to implant quality and familiarity. This supports findings from studies in Australia and Switzerland, where registry-derived revision data has been shown to guide and improve clinical decision-making at a national level [6, 10]. However, actual engagement with registry reports varied in our sample, especially among trainees. Notably, consultants valued registry data more highly and consulted it more frequently, suggesting a potential gap in training and awareness among junior doctors. Prior work by Varnum et al. has shown that increased registry use is associated with lower revision rates and greater standardisation in implant selection [6]. Investing in and developing INOR is therefore integral not only to improving data completeness and transparency but also to empowering surgeons with robust, evidence-based feedback on implant performance. A more comprehensive and actively utilised registry would support benchmarking, guide practice refinement, and may ultimately help reduce revision rates. This would in turn deliver both clinical and economic benefits for the Irish healthcare system.

At present, financial and clinical effectiveness data are not routinely integrated or presented together for orthopaedic surgeons in Ireland. While national joint registries provide robust information on implant survivorship and revision rates, implant cost data are typically managed through separate procurement systems and are not consistently available at the point of clinical decision-making. This separation may limit surgeons’ ability to make fully informed value-for-money decisions and may partly explain why cost was ranked as a secondary factor despite reasonable levels of cost awareness.

Despite over half of respondents reporting at least approximate knowledge of implant costs, few rated cost as a top influencing factor. This is consistent with international trends, where even cost-aware surgeons prioritise familiarity and perceived performance over price [5, 12]. In our study, very few registrars reported knowing the exact cost of implants, underscoring a lack of cost exposure during training. This is concerning given that surgeon-led implant choice represents a major determinant of total procedural cost [13]. Educating doctors on health economics has been shown to improve cost-consciousness and resource stewardship, both of which are increasingly recognised as core competencies in medical education [14, 15]. Given that total hip and knee arthroplasties are among the most commonly performed and resource-intensive procedures in the Irish healthcare system, integrating health economics into orthopaedic training is essential to ensure responsible use of finite resources [16, 17]. A more cost-literate surgical workforce could help bridge the gap between clinical autonomy and value-based care, ultimately enhancing sustainability and patient outcomes.

However, cost awareness and value-based decision-making are unlikely to be achieved without appropriate autonomy and early involvement in implant selection. A significant gradient was observed in implant selection autonomy between consultants and registrars. Consultants, particularly in the private sector, reported high levels of independence, while registrars frequently indicated limited or no input. These findings are consistent with results from oral and maxillofacial surgery, where junior doctors often operate under strict consultant-led implant protocols, limiting opportunities for decision-making and cost considerations [11]. This may perpetuate a cycle in which autonomy is delayed until late career stages, further reinforcing entrenched preferences and hindering the development of cost-conscious practice from an early stage.

Beyond the immediate issue of prosthesis selection, our findings highlight several important implications for orthopaedic practice and training. Orthopaedic surgery is increasingly expected to align with broader health system goals of sustainability and value-based care. Successful protocol-driven innovations such as Virtual Fracture Clinics in fracture management have demonstrated that high-quality outcomes can be maintained while reducing unnecessary hospital attendances and resource use [18], while Day of Surgery Admission protocols and day-case arthroplasty pathways have shown cost savings without compromising outcomes [19, 20]. These examples illustrate orthopaedics’ capacity to adopt evidence-based models that enhance efficiency. Applying similar structured approaches to implant selection, through integration of registry-derived outcomes with procurement and cost data, may support more transparent and value-based decision-making. In parallel, targeted education for surgeons and trainees in implant value, health economics, procurement processes, and registry interpretation is essential. Prior studies have demonstrated that structured feedback and formal training in cost-awareness can improve purchasing decisions without adversely affecting clinical outcomes [21, 22]. A coordinated multidisciplinary team approach, involving theatre staff, allied health professionals, and procurement specialists, may further support consistent, evidence-informed implant selection and continuity of care [23].

To ensure long-term sustainability, orthopaedic departments must cultivate a culture of ongoing clinical audit, economic accountability, and innovation. Leveraging data-driven decision-making, investing in continuous education, and supporting collaborative practice will be central to delivering safe, high-value arthroplasty care in increasingly resource-constrained environments.

### Strengths and limitations

This study’s strengths include its national scope and the inclusion of perspectives from both consultant orthopaedic surgeons and higher surgical trainees, providing a comprehensive overview of prosthesis selection practices across experience levels. The use of a structured, peer-reviewed survey instrument based on validated methodology enhances the study’s internal consistency and facilitates international comparison.

However, several limitations must be acknowledged. First, the sample may under-represent certain hospital groups or surgeons with lower arthroplasty volumes, potentially skewing results towards higher-volume centres or subspecialists. Second, terms such as “implant quality” and “departmental consensus” were not formally defined, which may have introduced variability in interpretation and response. Third, the voluntary nature of survey participation raises the possibility of response bias. Surgeons with strong views or preferences regarding specific implants may have been more motivated to participate, while those with less interest in arthroplasty or in procurement decision-making may have opted not to respond. Similarly, trainees not intending to pursue a career in arthroplasty may have been less inclined to complete the survey, which could influence the representativeness of junior surgeon responses.

Lastly, the study captures self-reported attitudes and preferences, which may not fully align with actual clinical practice. Prior research has highlighted discrepancies between stated implant preferences and observed behaviour, further underscoring the complexity of decision-making in orthopaedic surgery [24].

## Conclusion

This study identifies familiarity, implant quality, and revision rate as the key drivers of prosthesis selection among Irish orthopaedic surgeons. Autonomy is high among consultants but limited among registrars, with implications for training and long-term variation. Cost, while known by many, remains a secondary consideration. These findings suggest that surgeon education, registry engagement, and procurement policy reform could contribute to more consistent, evidence-informed implant choices.

## Data Availability

Not applicable.
